# Using Bionics to Restore Sensation to Reconstructed Breasts

**DOI:** 10.3389/fnbot.2020.00024

**Published:** 2020-05-08

**Authors:** Stacy T. Lindau, Sliman J. Bensmaia

**Affiliations:** ^1^Department of Obstetrics and Gynecology and Medicine-Geriatrics, The University of Chicago, Chicago, IL, United States; ^2^Department of Organismal Biology and Anatomy, Division of Biological Sciences, The University of Chicago, Chicago, IL, United States; ^3^Grossman Institute for Neuroscience, Quantitative Biology, and Human Behavior, Division of Biological Sciences, The University of Chicago, Chicago, IL, United States

**Keywords:** embodiment, tactile feedback, bionics, breast, mastectomy, sensation

## Abstract

Mastectomy often leads to a complete desensitization of the chest, which in turn can give rise to diminished sexual function and to disembodiment of the breasts. One approach to mitigate the sensory consequences of mastectomy is to leverage technology that has been developed for the restoration of sensation in bionic hands. Specifically, sensors embedded under the skin of the nipple-areolar complex can be used to detect touches. The output of the sensors then drives electrical stimulation of the residual intercostal nerves, delivered through chronically implanted electrode arrays, thereby eliciting tactile sensations experienced on the nipple–areolar complex. The hope is that the bionic breast will restore a woman’s sense that her breast belongs to her body so she can experience the pleasure of an embrace and derive the benefit of the sensual touch of her partner.

## Introduction

“…*one breast was removed and reconstructed, with nipple sparing, and the other was changed to ‘match it’*…*No one told me that I would lose erotic sensation, and I waited a long, sad time to realize that the sensation that was, for me, dominant in my sexual arousal would never return.*”

Cheryl Lilienstein, breast cancer survivor, commenting online to a New York Times article,1/29/2017

More than 2 million women were diagnosed with breast cancer globally in 2018, one in eight of these cases in the United States (Bray et al., 2018; Siegel et al., 2018). Of more than 3.8 million female breast cancer survivors in the United States today, about a third have undergone mastectomy and hundreds of thousands have had breast reconstruction procedures (Morrow et al., 2014; Kummerow et al., 2015; Wong et al., 2016). More than 100,000 women have one or both breasts removed each year. Since passage of the 1998 U.S. Women’s Health and Cancer Rights Act (WHCRA), which mandates insurance coverage for breast reconstruction after mastectomy, the number of women with mastectomy who choose to undergo breast reconstruction procedures has increased threefold (Kummerow et al., 2015). Additionally, 80% of a growing number of women in North America and Europe without breast cancer, but at elevated risk, elect reconstruction after prophylactic mastectomy (Cemal et al., 2013; Semple et al., 2013). Rates of breast reconstruction after mastectomy are high among women living in United States, Canada, and many European countries (Semple et al., 2013). Although rates are lower in low- and middle-income countries due to resource and access constraints, breast reconstruction procedures after mastectomy are performed around the globe (Anderson et al., 2008).

## The Role of Breast Sensation in Female Sexual Function

Loss of breast sensation after mastectomy is a prevalent, well-established, and distressing outcome for women and their partners that—despite best practice guidelines—is rarely addressed in the course of breast cancer care (Snell et al., 2010; Marshall and Galea, 2015; Flitcroft et al., 2017). Advances in breast preservation and reconstruction (including skin-sparing, nipple-sparing, deep inferior epigastric artery perforate, and neurotization techniques) aim to better preserve breast sensation to touch. However, these procedures typically have strict eligibility criteria, sensation outcomes are highly unpredictable, and normal breast sensation is rarely preserved (Magarakis et al., 2013; Zhou et al., 2018). Although surgical innovation continues to evolve (Peled and Peled, 2019), preservation of the distal branches of the intercostal nerves supplying the breast is typically infeasible for women with breast cancer due to concern about oncologic safety (Liebig et al., 2009). Breast transplantation has not been documented. However, transplantation requires immunosuppression, an unacceptable risk for a breast cancer patient, and would likely yield a poor sensory outcome. Although concern for sensory outcomes after mastectomy is growing, the majority of research paradigms in breast reconstruction after mastectomy have focused on aesthetic or cosmetic rather than functional outcomes.

Breast sensation is an important aspect of breast function for two reasons. First, the sense of touch is essential to embodiment or a person’s feeling that a body part belongs to her (Botvinick and Cohen, 1998). Patients seen at the University of Chicago Program in Integrative Sexual Medicine have said about their breasts after reconstruction: “They look great in a sweater, but they are dead to me” and “Aesthetically, they look awesome, but they do nothing for me.” These statements corroborate zoologist Stephen Wainwright’s maxim, especially apt for sense of touch: “Structure without function is a corpse” (Wainwright, 1988). The disconnect between body appearance and function can have deleterious consequences on overall physical, psychic, and social function. Second, breasts play an important role in female sexual function (Levin and Meston, 2006). From a patient with numb breasts after reconstruction: “When you don’t feel something and you know someone is touching them, it’s a turn-off.” Some women describe aversion to and avoidance of sex or even a feeling of anger, disgust, or dissociation during sexual contact with their numb breasts after mastectomy. Nipple–areolar complex sensation is an essential component of arousal and orgasm physiology for many people and their partners (Levin, 2006). Sexual dysfunction affects as many as three quarters of women with breast cancer and is at least partially attributable to loss of breast sensation (Ganz et al., 1999; Fobair et al., 2006; American Psychiatric Association, 2013; Raggio et al., 2014; Rojas et al., 2017).

## Preservation and Restoration of Sexual Function in Men With Cancer

In contrast to breast cancer care, medical decision-making for men with prostate cancer is routinely informed by evidence about sexual function outcomes associated with treatment options (American Urology Association, 2007). Preservation and restoration of sexual function in men with prostate cancer has been identified as an important patient-centered outcome. The effort to improve sexual function outcomes for men after prostate cancer (American Urology Association, 2007; Grondhuis Palacios et al., 2017; Elliott and Matthew, 2018; Girodet et al., 2019) has driven substantial surgical innovation over the last two decades, supported in part by the U.S. National Institutes of Health, and has produced significant improvements (Willis et al., 2012). In 2016, a man with penile cancer (a cancer type affecting < 0.001% of men) underwent a successful penile transplant. Cosmesis was not the only outcome of interest—the measure of success in this and a subsequent case also required restoration of urinary, reproductive, and sexual function (Grady, 2016; Merwe et al., 2017). Between 2014 and 2018 at least four successful penile transplants have been accomplished worldwide. These breakthrough efforts to preserve male sexual function, including in the context of penile cancer, both inspire and legitimize innovation to preserve breast function for the large and growing population of women with or at elevated risk of breast cancer (Grady, 2016; Merwe et al., 2017; McDaniels, 2018).

## Toward a Bionic Breast

One approach to restore function to reconstructed breasts is to apply bionic technologies that have been successful in sensitizing prosthetic hands. Sensitization of the prosthetic hand is achieved by electrical stimulation of residual nerves delivered through chronically implanted arrays of electrodes, which evokes sensations that are referred to the phantom hand (Saal and Bensmaia, 2015). The referred sensations are highly localized and repeatable, and their magnitude can be manipulated by modulating the amplitude or frequency of microstimulation (Graczyk et al., 2016). These phenomena can be exploited by connecting pressure sensors on the bionic hand to electrodes with somatotopically appropriate projection fields. For example, the pressure sensor on the index fingertip of the bionic hand drives stimulation though an electrode that evokes sensations on the index fingertip. Bionic hands endowed with artificial tactile feedback confer greater dexterity to users than do insensate ones (Ortiz-Catalan et al., 2014; Valle et al., 2018; George et al., 2019). Furthermore, the incorporation of artificial touch leads to a greater embodiment of the hand (Marasco et al., 2011; Schiefer et al., 2016; Page et al., 2018; Rognini et al., 2018; Valle et al., 2018) and restores some of the key psychosocial components of manual touch, including the ability to experience pleasure from touching a loved one (Graczyk et al., 2018, 2019; Cuberovic et al., 2019; George et al., 2019).

**FIGURE 1:**
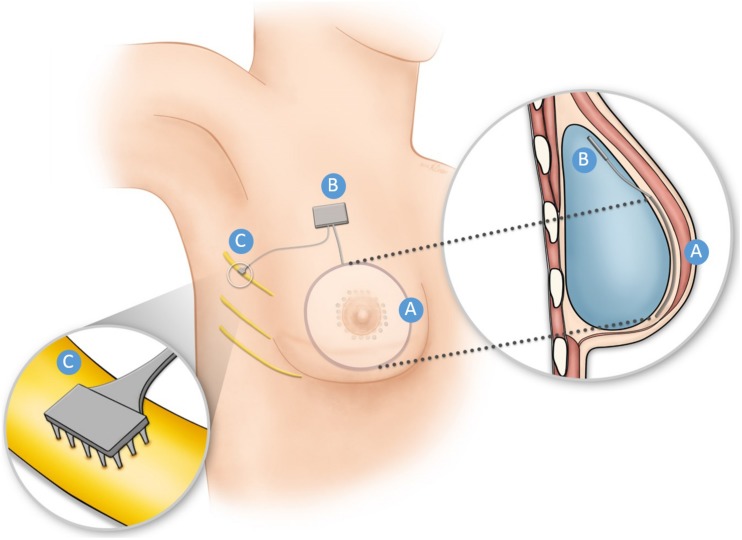
Schematic illustration of the Bionic Breast concept for a breast reconstructed using an implant. An array of pressure sensors **(A)** is placed under the reconstructed or tattooed nipple and the surrounding region. The output of the sensors triggered by pressure applied to the skin is converted into electrical stimulation pulse trains via a hermetically sealed electronic circuit **(B)**, shown here to be encased in the breast implant. The electrical stimulation pulse trains are delivered through one or more electrode arrays **(C)** implanted around or in the residual intercostal nerve(s) that innervated the nipple areolar complex before mastectomy. Nerve stimulation results in a sensation projected to the nipple areolar region. Much of this technology has already been developed to restore sensation to bionic hands.

This strategy can be straightforwardly applied to reconstructed breast. An array of sensors would be implanted under the skin of the breast centered on the nipple (Figure 1), an application for which recent advances in flexible sensory technology can be leveraged (Boutry et al., 2019; Niu et al., 2019; Ruth et al., 2019). The output of the sensors would drive electrical stimulation through arrays implanted in residual intercostal nerves III–VI, which carry sensory signals from the breast. Various technologies have been developed to provide electrical interfaces with the peripheral nerves, any one of which would be appropriate for this application (Saal and Bensmaia, 2015). The sensory encoding algorithms—which convert patterns of sensor output into trains of electrical stimulation—would be implemented in a hermetically sealed electronic circuit that would be lodged in the implant or just under the skin. Such an implantable circuit—with the appropriate inputs and outputs—has already been developed for bionic hands (Peterson et al., 2016). The spatial extent of the sensor array under the reconstructed breast will depend on which intercostal nerves are implanted with stimulating arrays. Minimally, the array will span the nipple–areolar complex, but may cover the entire breast if all four intercostal nerves are accessed. Given the current state of technology, the principal bottleneck in the spatial resolution of artificial touch is set by the electrical interface with the nerve, not the sensors, so sensor pitch will not be a major design specification for the sensor sheets.

The current consensus is that the tactile sensibility of the breast is similar to that of the hand, so sensory encoding models developed for the hand (Saal et al., 2017; Okorokova et al., 2018) can likely be applied to the breast, accounting for differences in the relative densities of the different subtypes of tactile nerve fibers. A limitation of this approach is that thermal sensation—key to affective and sexual touch—cannot be restored through electrical stimulation because thermoreceptive nerve fibers are small and unmylienated and thus insusceptible to electrical stimulation. Similarly, nerve fibers implicated in affective touch—c-tactile fibers—will not be activated by the electrical interface. With these caveats, restoration of tactile sensation alone is liable to have a major impact on the sexual and psychosocial function of the breast, based on its documented impact on manual function.

The U.S. National Cancer Institute has invested in the Bionic Breast Project, a new, interdisciplinary program of research that is laying the foundation of basic knowledge—including development of subjective and objective measures of female breast function—to apply bionic technologies to restoration of breast function after mastectomy. In addition to improving outcomes for women with breast cancer, this work could hold promise for improved outcomes after reconstruction for traumatic breast injury and for people undergoing elective breast surgery, for example, for gender affirmation.

## Discussion

Breakthroughs in restoration of hand and penile function, including for intimate and sexual function, have laid the scientific and moral foundations for the preservation and restoration of breast function in the millions of women affected by breast cancer worldwide. Clinical guidelines in the United States, United Kingdom, and Australia recommend women undergoing mastectomy be offered the option of breast reconstruction (National Breast Cancer Centre, 2001; Harnett et al., 2009). Advocating to the United States Congress about the WHCRA in 1998, a senator expressed his outrage at the medical director of an insurance company who argued that, because the breast “is not a bodily function,” its replacement is not medically necessary (Senate, 1998). Sensitization of the reconstructed breast will promote its embodiment and mitigate the sensory component of mastectomy-induced sexual dysfunction. Preservation and restoration of breast sensation, along with other aspects of breast function are attainable, imperative, and well within the coverage bounds for breast reconstruction mandated by U.S. WHCRA (Centers for Medicare & Medicaid Services, 2013).

## Author Contributions

SL conceived of the idea. SB contributed technically to the concept. SL and SB wrote and edited the manuscript.

## Conflict of Interest

The authors declare that the research was conducted in the absence of any commercial or financial relationships that could be construed as a potential conflict of interest. The University of Chicago has filed a provisional patent related to the work described here.
